# Tumor educated platelet: the novel BioSource for cancer detection

**DOI:** 10.1186/s12935-023-02927-5

**Published:** 2023-05-11

**Authors:** Shanshan Ding, Xiaohan Dong, Xingguo Song

**Affiliations:** 1 Department of Clinical Laboratory, Shandong Cancer Hospital & Institute, Shandong First Medical University & Shandong Academy of Medical Sciences, Jinan, Shandong PR China; 2 Department of Laboratory Medicine, Affiliated Hospital of Jining Medical University, Jining, Shandong China

**Keywords:** Tumor educated platelet, Small nuclear RNA, Tumor biomarker

## Abstract

Platelets, involved in the whole process of tumorigenesis and development, constantly absorb and enrich tumor-specific substances in the circulation during their life span, thus called “Tumor Educated Platelets” (TEPs). The alterations of platelet mRNA profiles have been identified as tumor markers due to the regulatory mechanism of post-transcriptional splicing. Small nuclear RNAs (SnRNAs), the important spliceosome components in platelets, dominate platelet RNA splicing and regulate the splicing intensity of pre-mRNA. Endogenous variation at the snRNA levels leads to widespread differences in alternative splicing, thereby driving the development and progression of neoplastic diseases. This review systematically expounds the bidirectional tumor-platelets interactions, especially the tumor induced alternative splicing in TEP, and further explores whether molecules related to alternative splicing such as snRNAs can serve as novel biomarkers for cancer diagnostics.

## Introduction

**Fig. 1:**
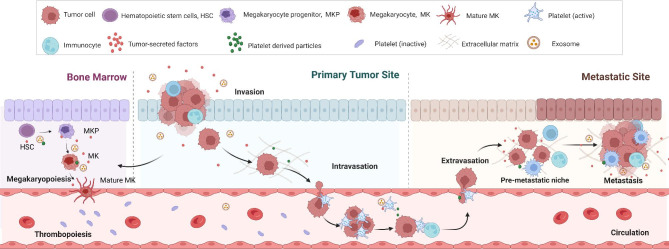
**The crosstalk between cancer and platelets.** Tumor educates platelets: tumor can induce platelet activation, aggregation, and release of platelet-derived substances in circulation, and promote thrombocytosis via influencing megakaryopoiesis in bone marrow; Platelets support tumor growth and metastasis: platelets stimulate tumor angiogenesis and vascular remodeling, protect CTCs from shear forces and evade immune surveillance, and recruit stromal cells to facilitate the establishment of metastatic niches and promote the metastasis (MKP: megakaryocyte progenitor; MK: megakaryocyte; HSC: Hematopoietic stem cells)

Platelets, the most abundant anucleate cells except red blood cells in the circulation, originate from megakaryocytes in the bone marrow with a short average lifespan of 7 days [1]. Besides its role in hemostasis, platelets also play an important role in tumorigenesis and tumor progression [2]. Platelets stimulate tumor angiogenesis and vascular remodeling, protect CTCs from shear forces and evade immune surveillance, and recruit stromal cells to facilitate the establishment of metastatic niches and promote the metastasis. On the other point of view, tumor can also “educate” platelets. It induces platelet activation, aggregation, and release of platelet-derived substances in circulation, and promote thrombocytosis via influence megakaryopoiesis in bone marrow (Fig. 1). During the bidirectional tumor-platelet interactions, platelets systematically and locally respond to cancer, as well constantly absorb and enrich free proteins, nucleic acids, vesicles and particles [3, 4], leading to the alterations in their RNA and proteomics expression profiles [5, 6], thus termed “tumor educated platelets” (TEPs) [2].

The changes of TEPs profile represent a massive, concentrated biorepository of tumor-derived and bioactive molecules, indicating the potential of TEPs as specific biomarkers for cancer. Due to the short lifespan and the structure of platelet membrane, tumor-specified biosources and biomolecules are enriched in TEPs and protected from circulating RNAase and other enzymes, thus contents in TEP are capable to reflect tumor bioactivity up-to-date, intensive, and dynamically, playing the crucial roles in cancer detection and progression monitoring including colorectal carcinoma (CRC), glioblastoma, non–small cell lung cancer (NSCLC), prostate cancer, and etc. Platelet lacks a nucleus; no genomic DNA is available for transcription of new RNA molecules. Quantification of platelet RNA demonstrates approximately ~ 2.2 fentogram of RNA in one single platelet, but 20–40 times in younger, reticulated platelet [7, 8], indicating a variety of RNA regulatory biological processes, such as RNA splicing.

RNA splicing in TEPs can be induced by external signals (such as platelet surface receptor activation), or in response to signals released by tumor microenvironment, resulting in highly dynamic mRNA repertoires with potential tumor diagnostic applications [9]. Platelets contain many proteins associated with the spliceosome and small nuclear RNAs (snRNAs) to form small nuclear ribonucleoproteins (snRNPs) [10, 11]. SnRNAs including U1, U2, U4, U5, U6 are not merely the basal factors ubiquitously expressed in all cells since they are required for the guidance of pre-mRNA splicing [12], whereas they are extremely variable across a wide range of biological conditions [13]. The endogenous alterations in TEP snRNAs can modulate alternative splicing [14], thereby contributing to the alternation of TEP mRNA profile significantly. Although TEP mRNA has been well-recognized as the promising biomarkers for liquid biopsy in various tumors in recent years [15], it is generally uninformed about the regulation of TEP alternative splicing and its role in cancer diagnostics. This review systematically expounds the bidirectional tumor-platelet interactions, especially the tumor induced alternative splicing in TEP, and further explores whether molecules related to alternative splicing such as snRNAs can serve as novel biomarkers for cancer diagnostics.

## The interactions between platelets and tumor

### Tumor cells changes platelets

#### Structure basis of tumor-platelet direct interactions

**Fig. 2:**
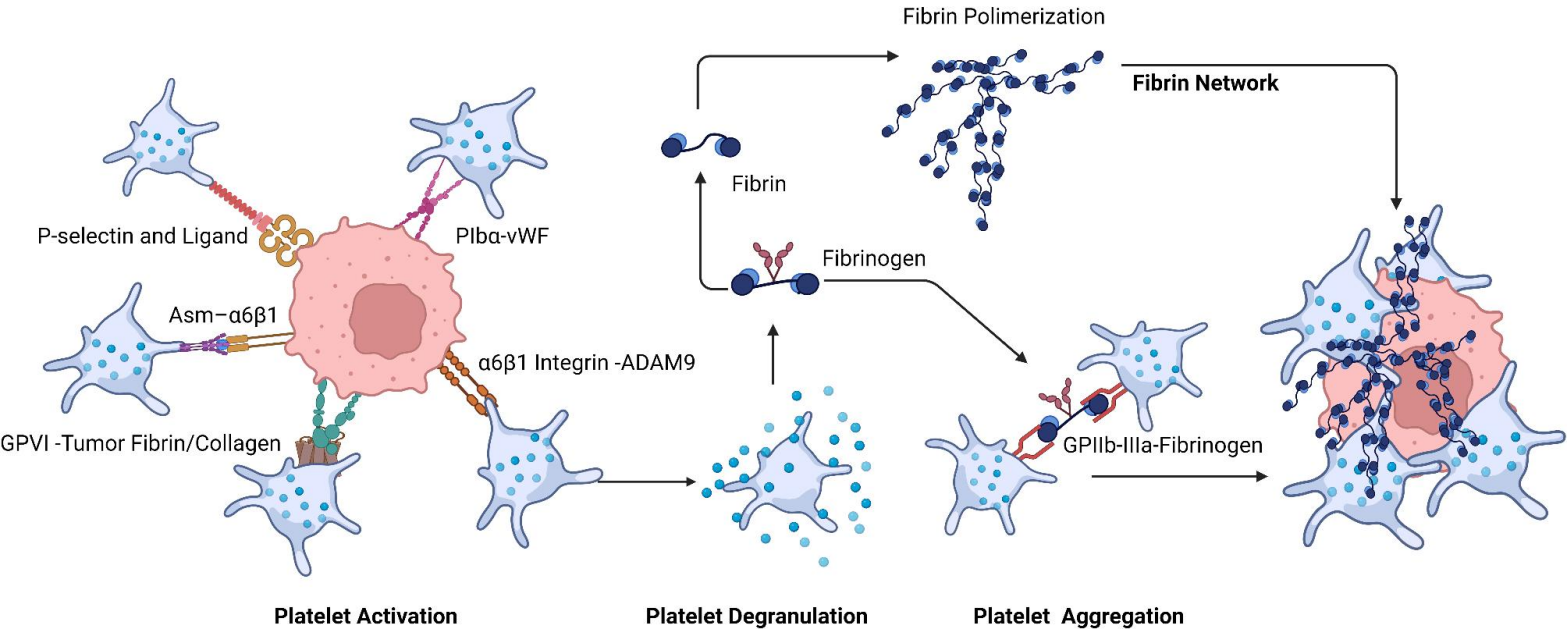
**Structure basis of tumor-platelet interaction and molecular mechanisms of TCIPA.** Direct surface receptor binding and extracellular protein-mediated receptor bridging are the structure basis of tumor-platelet interactions (left); The interactions trigger platelet activation and degranulation, in turn aggregation (TCIPA) dependent on GPIIb-IIIa and fibrin network (right)

Direct surface receptor binding and extracellular protein-mediated receptor bridging were the structure basis of tumor-platelet interactions [16–18] (Fig. 2). Numerous studies have investigated the targeting direct molecule contacts, including platelet GPIIb-IIIa (also called αIIbβ3 integrin)-plasma fibrinogen or fibronectin - tumor αVβ3 integrin [19–21]; platelet GPIbα - tumor Von Willebrand Factor (vWF) [22–24]; platelet GPVI - tumor fibrin and/or subendothelial collagen [25, 26]; platelet α6β1 integrin-tumor ADAM9 [27]; platelet acid sphingomyelinase (Asm) – tumor α6β1 integrin [28, 29]; platelet CLEC-2-tumor podoplanin [30–32]; and platelet P-selectin-tumor P-selectin ligand [33–35]. These platelet receptors and their ligands mediate tumor growth, metastasis and direct tumor-platelet interactions.

#### Tumor cells induced aggregation

Additionally, tumors can induce platelet aggregation by directly interacting with platelets. Once tumor cells leave the primary tumor site and enter the blood circulation, they directly lead to platelet activation and aggregation, whereby platelets protect tumor cells from immune cell-induced cell death [36], a phenomenon known as “Tumor cell-induced platelet aggregation (TCIPA) [37, 38] " (Fig. 2). In this way, TCIPA can trigger platelets to release a large amount of pro-tumorigenic factors to fuel tumor growth [39]. Current studies have suggested that TCIPA mainly works through the following pathways: (i) tumor cell-platelets interactions result in the formation of small amounts of thrombin, which may trigger platelet activation and aggregation, (ii) fibrinogen binding to integrin αIIbβ3 and fibrin formation can mediate platelet aggregation, and (iii) tumor cells cause some ATP/ADP to be released from dense granules, and the release of ADP stimulates P2Y12 receptors that are necessary for platelet aggregation [37, 40].

#### Tumor cells promote thrombocytosis

As early as the 19th century, studies first reported the relationship between thrombocytosis and tumors, which was common in tumor patients [41], whereas the interaction of platelets and cancer cells formed a positive feedback cascade to potentiate the effect. It was reported the increased platelet count was associated with poor overall and/or progression-free survival and revealed as predictors of a variety of cancers [42, 43], including lung cancer [44], ovarian cancer [45], gastric cancer [41], colorectal cancer (CRC) [46] and breast cancer (BrCa) [47]. Platelet count might also be an effective biomarker for monitoring disease recurrence and predicting treatment response in patients with epithelial ovarian cancer (EOC) [48], and rectal cancer [49]. Meanwhile, other platelet-associated clinical laboratory indexes including platelet to lymphocyte ratio (PLR) [50–53], platelet distribution width to platelet count ratio [54, 55], platelet to albumin ratio [56], and red cell distribution width to platelet count ratio [57] were also associated with poor progression and were shown to predict of a variety of cancers, as summarized in Table 1.

**Table 1:** Platelet-associated clinical laboratory indexes as prognostic biomarkers of tumors

Clinical Laboratory Indexes		Functions		Tumor types	References
platelet count	predicting prognosis			NSCLC, lung, gastric, ovarian, breast, colorectal cancers, hypopharyngeal squamous cell carcinoma, esophageal squamous cell cancer (ESCC), renal cell carcinoma	[41, 42, 44, 46, 47, 151, 152, 153, 154, 155, 156]
	monitoring the disease recurrence and predicting treatment response			EOC, rectal cancer	[48, 49]
	predicting lymph node metastasis			NSCLC	[157]
PLR	predicting prognosis			NSCLC, lung, breast, gastric, bladder, metastatic colorectal cancers	[50, 51, 52, 53, 158]
	monitoring the disease recurrence and predicting treatment response			TNBC	[159]
	predicting survival outcomes			rectal, cervical cancers	[160]
	predicting lymph node metastasis			breast cancer	[161]
platelet distribution width to platelet count ratio	predicting prognosis			breast, serous ovarian cancer	[54, 55]
platelet to albumin ratio	predicting prognosis			NSCLC	[56]
red cell distribution width to platelet count ratio	predicting prognosis			breast cancer	[57]
PMPs	predicting prognosis			breast cancer, epithelial ovarian cancer	[162, 163]
	predicting survival outcomes			prostate cancer	[164]
CD40L	predicting prognosis			gastric cancer, colorectal cancer	[65, 165]
	predicting survival outcomes			cancer-associated VTE	[59, 166]
P-selectin	predicting prognosis			colorectal cancer, cancer-associated VTE	[60, 66, 167, 168)
TF	predicting prognosis			cancer-associated VTE	[61, 169, 170]

**Fig. 3:**
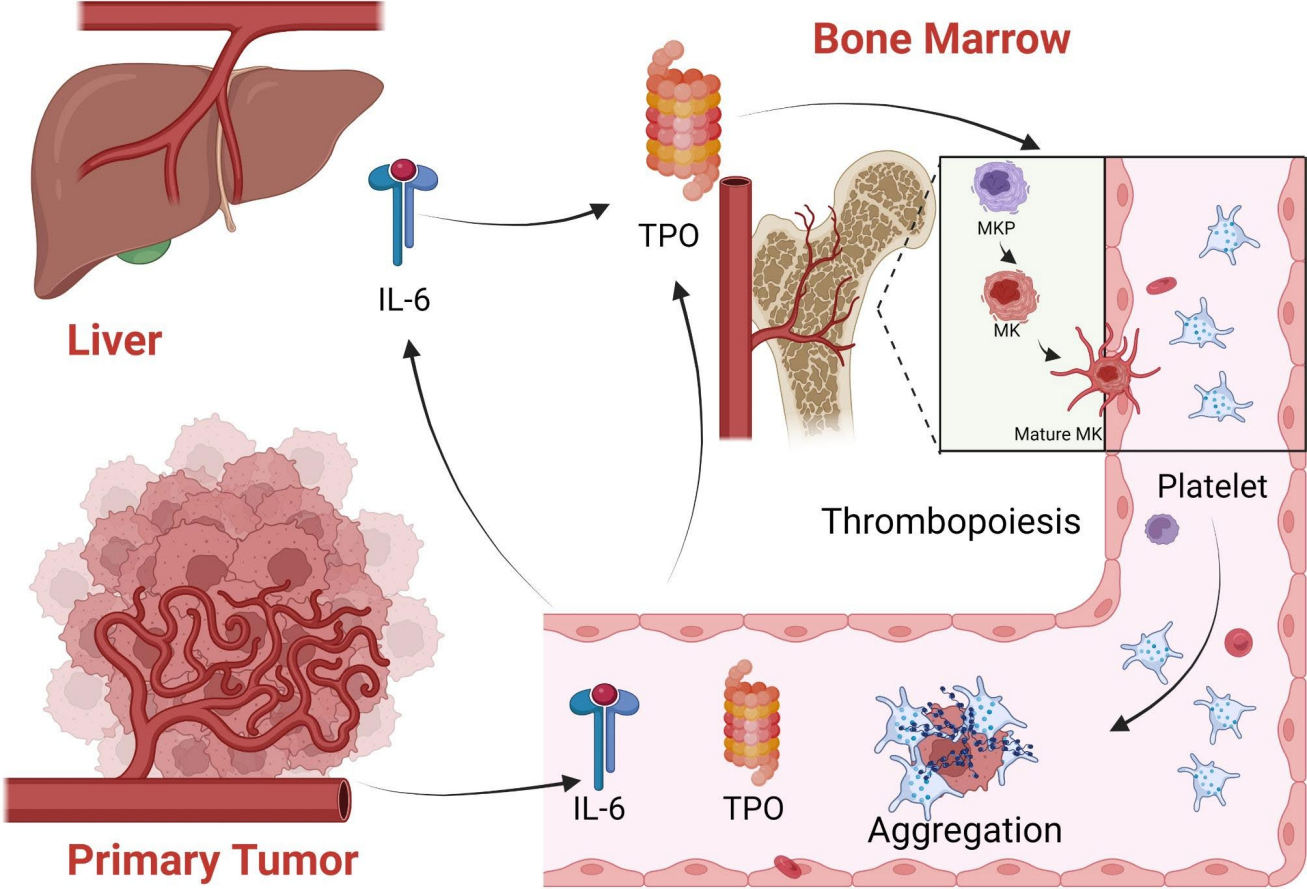
**Mechanisms of cancer-associated thrombocytosis.** Primary tumor cells secret TPO, or IL-6 which can accelerate TPO production in the liver. TPO can stimulate differentiation, proliferation and maturation of megakaryocytes in the bone marrow, as well as platelet production (TPO: thrombopoietin; IL-6: interleukin-6; MKP: megakaryocyte progenitor; MK: megakaryocyte)

Several evidence had revealed the main molecular mechanisms of thrombocytosis (Fig. 3), including (i) tumor cells secret thrombopoietin (TPO), or interleukin-6 (IL-6) which can accelerate TPO production in the liver. TPO in turn stimulates thrombopoiesis in bone marrow [45]; (ii) TPO can stimulate differentiation, proliferation and maturation of megakaryocytes; (iii) tumor cells can accelerate platelet destruction and then induce compensatory thrombocytopenia; and (iv) malnutrition, chronic blood loss from tumor depletion, and myeloproliferative diseases can also cause thrombocytosis [58].

#### Tumor cells promote production of platelet-derived substances

Moreover, cancer patients also present with increased expression levels of platelet-derived substances in the circulation, including CD40 ligand (CD40L) [59], P-selectin [60], tissue factor (TF) [61] and platelet-derived microparticles (PMPs) [62, 63]. The platelet activation markers CD40L and P-selectin play immunosuppressive effect and are used as indicators of disease progression in cancer or cancer-associated venous thromboembolism (VTE) patients [64–66]. It has been shown that aggressive tumors are correlated with higher levels of platelet microparticles. For example, miRNA-223 delivered by platelet-derived microparticles is significantly increased in patients with NSCLC. Tumors also induce platelet degranulation and phenotype changes in cancer patients by increasing the secretion of pro-angiogenic proteins, such as vascular endothelial growth factor (VEGF). Altogether, these studies have demonstrated cancer-activated platelets induce a procoagulant environment, providing early biomarkers for cancer screening (Table 1).

### Platelets support tumor growth and metastasis

#### Platelets stimulate tumor angiogenesis and vascular remodeling

Platelets stimulate tumor angiogenesis through multiple mechanisms, resulting from the complex interplay between cancer cells and platelets in regulating tumor neovascularization [67]. This intercellular communication depended on the secretion of platelet α-granules, the treasure trove of the angiogenic factors in the tumor microenvironment containing VEGF and cytokines [68, 69]. In addition, the important angiogenic agents such as fibroblast growth factor (FGF), platelet-derived growth factor (PDGF) and PMPs also affect angiogenesis and indirectly enhance vessel formation [67, 69]. Thus, as an important source of angiogenesis-related factors in circulation, platelets act as “first responders” across the full spectrum of cancer progression, they and their products stimulate stroma release, promoting angiogenesis and chemotaxis [70].

In addition to regulating angiogenesis, platelets can also regulate vascular integrity, relying on the secretion of angiopoietin-1 (ANGPT1) and serotonin of α-granules, thereby promoting endothelial integrity and barrier function in primary tumors [71, 72]. While angiopoietin-2 (ANGPT2) secreted by VEGF activated endothelium could inhibit ANGPT1 competitively and destabilize vessel assembly [73]. Therefore, the stability of tumor vessel depends on the balance between the tumor and platelet-derived granules. In lymphatic vessels, platelets maintained the stability of blood-lymphatic system to support angiogenesis and tumor growth [74, 75]. Platelets might also reduce immune cells infiltration by regulating vascular integrity, reducing tissue damage by protecting tumor cells from assault of natural killer cells (NK cells) [76, 77]. Thus, platelets exhibit pro-tumorigenic functions, which directly or indirectly promote tumor growth by regulating tumor angiogenesis and vascular integrity.

#### Platelets support tumor invasion and metastasis

Invasion and metastasis are important features of tumorigenesis and development, and platelets also play an important role in this process. As the first cell to encounter tumor cells, it interferes with immune system surveillance to protect circulating tumor cells [78]. Upon the migration and colonization of invasive tumor cells in the blood, platelets can improve their survival and support metastatic dissemination [77]. Platelet-derived TGF-β is complexed with glycoprotein A repetitions predominant (GARP) protein to induce both NK cells and T cells anergy [79], while thrombin involved in platelet-bound GARP cleavage and the liberation of active TGF-β supports cancer immune evasion [80].

Furthermore, platelet-tumor interactions support the occurrence of epithelial-mesenchymal transformation (EMT)-like events and metastasis [81]. Platelets release EMT inducers and growth factors to shift epithelial-like phenotype to mesenchymal-like phenotype [82, 83]. Subsequently, platelet-associated cell adhesion molecules (CAMs), including integrin, P-selectin, immunoglobulin superfamily (IgSF) member glycoprotein VI (GPVI), etc. [26, 84, 85], can mediate adhesion and communication between platelets and the extracellular matrix (ECM) and among platelets to promote tumor metastasis [86]. Finally, tumor-platelet agglomerates support intravascular arrest of cancer cells via P-selectin, accelerating extravasation to distant organs [87]. In the process, platelet-secreted chemokines (like CXCL5 and CXCL7) [88], growth factors (like VEGF, PDGF, and TGF-β)[89], and PMPs-derived miRNA [90] support the proliferation, formation of pre-metastatic nitch and seeding of metastatic tumor cells. Therefore, platelets play a key role in tumor cells proliferation progression, anoikis resistance, extravasation and metastatic seeding.

To sum up, platelets are involved in the whole process of tumorigenesis and tumor development (Fig. 1). Benefit from their closed membrane structure, platelets can completely preserve the biological information of tumor sources and isolate bioactive molecules in the circulation. For these reasons, the substances carried by platelets have great potential to become tumor biomarkers (Tables 2 and 3).

**Table 2:** TEP RNA families in various tumors

RNA families	TEP biomarkers	Functions	Tumor types	References
messenger RNA (mRNA)	ITGA2B, EGFRvIII, PCA3, MAX, MTURN, HLA-B, ACIN1, TIMP1, TPM3, AKT, PI3K, RhoA, CTNNB1, SPINK1	tumor diagnosis	NSCLC, prostate, lung, colon, breast cancer, glioblastoma, HCC	[93, 98, 100, 171, 172, 173, 174, 175, 176]
	KLK2, KLK3, FOLH1, NPY, MAX, MTURN and HLA-B	predicting treatment response	prostate, lung cancer	[96, 100]
microRNA (miRNA)	miR-34c-3p, miR-18a-5p	tumor diagnosis	nasopharyngeal carcinoma (NPC)	[177]
small nuclear RNA (snRNA)	U1, U2, U5	tumor diagnosis	lung cancer	[134]
small nucleolar RNA (snoRNA)	SNORD55	tumor diagnosis	NSCLC	[178]
circular RNA (circRNA)	circNRIP1	tumor diagnosis	NSCLC	[179]
long noncoding (lncRNA)	lincGTF2H2-1, RP3-466P172, and lnc-ST8SIA4-12	tumor diagnosis	lung cancer	[180]
antisense RNA (asRNA)	MAGI2-AS3 and ZFAS1	tumor diagnosis	NSCLC	[172]

**Table 3:** TEP-derived proteins in various tumors

TEP-derived proteins	Functions	Tumor types	References
VEGF, PDGF, PF4, TSP1 and TGF-β1	tumor diagnosis	colon, breast cancer	[181, 182]
PDGF, TGF-β1	predicting prognosis	HCC	[97]
EML4-ALK rearrangements	predicting treatment response	NSCLC	[183]
platelet proteome	tumor diagnosis	pancreas, ovarian cancer	[184, 185]

## Alterations and mechanisms of platelet RNA profiles in tumor

### Platelet mRNA expression profiles can serve as tumor biomarkers

mRNA is the most studied type of RNA in platelets, about one-third of all human genes (~ 5000–9000 genes) mRNAs have been identified within platelets [91, 92]. Previous studies have illuminated the diagnostic value of platelet mRNA signatures as the non-invasive biomarkers for predicting tumorigenesis and monitoring tumor progression, including CRC [93], lung cancer [94], NSCLC [95], prostate cancer [96], liver cancer (hepatocellular carcinoma, HCC) [97] and etc.

Best et al. prospectively isolated, amplified, and sequenced TEP mRNA profile between healthy donors and cancer patient platelets, 5,003 differentials were identified. Using this readout, they were able to distinguish patients with localized and metastatic tumors from healthy individuals with 96% accuracy [2]. Using the R language WGCNA package, platelet RNA profiles of CRC patients and healthy donors were screened for potential biomarkers for cancer diagnostics. It was found that TIMP1 mRNA in platelets increased for tumor patients, possessing the promising diagnostic performance much higher than CEA and CA199 [93]; Besides, platelet ITGA2B levels were significantly higher in NSCLC patients than in all controls, and the combination of ITGA2B, CEA and stage could predict the overall survival [98]; A similar phenomenon was observed in a pan-cancer study, where platelets mRNA expression profiles were significantly different between tumor patients and healthy volunteers. Platelet profiles were not only suitable for cancer diagnosis, but also correctly identified the primary origins of pan-cancer. In many cases, they could accurately predict tumor gene mutation status, including MET, HER2, KRAS, EGFR or PIK3CA mutations [99]. Calverley et al. also demonstrated that they could distinguish patients with HER2 amplified, PIK3CA mutant or triple-negative BrCa (TNBC) and NSCLC patients with MET overexpression, although the low levels of these mutant biomarkers needed to be considered [99]. Our previous study also demonstrated significant changes in platelet mRNA expression profiles in lung cancer patients [100]. A total of 1306 mRNAs with the differential expression were identified, among which MAX, MTURN, UQCRH and HLA-B were significantly upregulated and correlated with ‘‘favorable’’ first chemotherapy response, thus providing a noninvasive marker to predict first chemotherapy response.

### Splicing is the major regulatory mechanism for TEP mRNA expression

Although mature platelets are anucleate, they still retain endogenous pre-mRNAs inherited from the transcription of nuclear DNA in the megakaryocyte as well exploit functional spliceosome [101]. Once activated by external signals, such as activation of platelet surface receptors and lipopolysaccharide-mediated platelet activation, these transcripts can be specifically spliced into mature mRNA and translated into thousands of different proteins [102]. RNA splicing is closely related to changes in platelet mRNA profiles, and analysis demonstrated that pre-mRNA splicing might occur during platelet activation [103]. For example, interleukin-1β (IL-1β) was spliced into mature mRNA transcripts, resulting in the synthesis of IL-1b proteins in response to cellular activation in quiescent platelets [101, 104, 105].

**Fig. 4:**
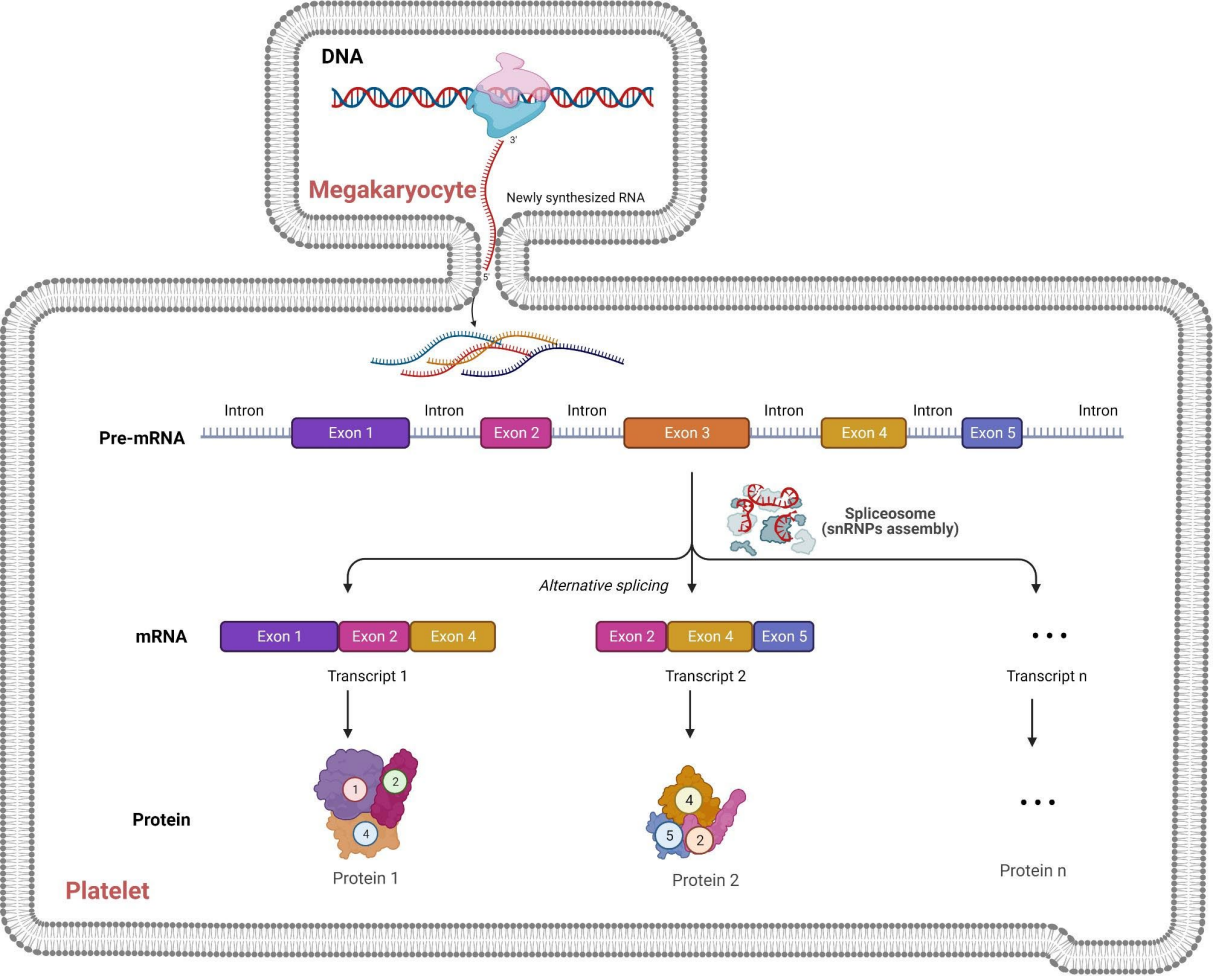
**Platelets exploit a functional spliceosome for pre-mRNA splicing.** Megakaryocytes sort distinctive RNA molecules into proplatelets during thrombopoiesis. Pre-mRNAs contain exons and introns and are processed by U snRNPs that make up the spliceosome. Platelet spliceosomes alternatively excise introns from pre-mRNA, yielding a mature message that is translated into protein

Aberrant RNA splicing is an underlying highly conserved process, occurring in > 95% of human multi-exon genes [106]. A Pan-Cancer study have found an average of 20% more alternative splicing in tumors than in corresponding healthy tissues [107]. Platelets may also undergo queue-specific splice events in response to signals released by cancer cells and tumor microenvironment [102]. The specific splice events can provide platelets with a highly dynamic mRNA repertoire in patients with different types and organs of tumors, with potential applicability to cancer diagnosis and tumor origin tracking [95, 99]. Previous research detected the differential expression of spliced RNAs in NSCLC patients based on the intron-spanning read count analysis. They identified 1,625 spliced platelet genes with significantly different spliced levels (698 genes with enhanced splicing in platelets of NSCLC patients and 927 genes with decreased splicing in platelets of NSCLC patients). The most significantly enriched spliced RNAs in TEPs included CFL1, ACOT7, and ARPC1B, whereas DDX5, RPS5, and EEF1B2 were decreased [95]. Therefore, a large number of changes in platelet splicing behavior during platelet activation are undoubtedly one of the main reasons for the changes in platelet mRNA expression profiles (Fig. 4).

## TEP snRNAs as novel biomarker in cancer detection

### snRNPs dominate RNA splicing

Chemical reactions of pre-mRNA splicing in platelets occur only after the pre-mRNA assembles into the functional spliceosome, a multi-component complex termed as snRNPs composed of U1, U2, U4, U5, U6 snRNAs and their associated protein components [10, 11], including a protein-only NineTeen Complex (NTC) and a number of accessory proteins [108, 109]. It has been shown that platelets contain many spliceosome-associated proteins, including U1 70 K, U2AF, SRm160, SMN, and SF2/ASF [7], as well as snRNAs, which direct the accurate removal of intronic sequences from pre-mRNAs.

During spliceosome assembly, snRNAs and splicing factors recognize and interact with the pre-mRNA consensus sequences, facilitating and specifying the transesterification reactions [110]. Their main process in the spliceosome complex is that U1 and U2 snRNPs are responsible for recognizing the 5′ splice site and branchpoint upstream of the 3′ splice site, and U4/U6.U5 tri-snRNP is added to the spliceosome before rearrangements, guiding U6 snRNP to catalyze the actual splicing reaction [13, 111]. In addition to U1, U2, U4, U5, and U6 snRNPs (major), other minor spliceosome snRNP species (U11, U12, U4atac, U5atac and U6atac) are also involved in splicing a minor class of introns [112, 113]. Eventually, introns are removed, and protein-coding segments known as exons are spliced together to form mRNAs [114, 115]. It was previously thought that spliceosome components were only present in nucleated cells [116], but later it was reported that anucleate platelets also exploit the functional spliceosome inherited from megakaryocytes during thrombopoiesis [101]. More Importantly, snRNAs are not merely the basal factors ubiquitously expressed in all cells [12], whereas they are extremely variable across a wide range of biological conditions [13].

### Alternation of snRNAs regulate alternative splicing in cancer

Recently, snRNPs have been shown to act as regulatory molecules to mediate cancer processes through alternative splicing [117, 118]. It can directly or indirectly affect too many molecular targets, thereby regulating cis-acting elements, transacting factors, or pre-mRNA transcription at multiple levels [119]. In particular, endogenous variation at snRNA levels leads to widespread differences in alternative splicing. Studies have shown that snRNA dysregulation shapes the transcriptome of breast cancer [13], exhibiting subtype-specific dependence on the abundance of different snRNAs [120, 121]. For example, the HER2 subtype shows high levels of U1 and U5A, while triple-negative samples have high abundance of U6 or relatively low levels of U2 and U5A [122].

SnRNAs can also be subject to somatic mutations in addition to aberrant expression, which can alter the normal splicing process to drive heredity, dysplasia, and even tumorigenesis and cancer progression [123, 124]. For example, aberrant U1 snRNA (A > C somatic mutation at the third base of U1) has been reported in several tumor types, generating novel splice junctions and altering the splicing pattern of multiple genes.

### Alternation of platelet snRNAs

Multiple hypotheses exist regarding the source and mechanism of platelet snRNAs alterations. One hypothesis supposes RNA expression patterns are fluid throughout megakaryocyte development and platelet biogenesis [125, 126]. Alterations of platelet snRNAs are caused by RNA differential sorting mechanism of megakaryocytes [127]. In addition, an alternative source mechanism has recently been discussed, namely the ability of extracellular vehicles (EVs) to transmit snRNAs horizontally [9]. Circulating platelets can capture and store tumor-derived EVs from the periphery, and then obtain characteristic biological information, which is one of the main mechanisms of TEP.

It has recently been shown that megakaryocytes selectively sort RNAs into platelets rather than randomly, allowing only a fraction of RNAs transferred into platelets. This observation is supported by a recent study describing how megakaryocytes preferentially sorted matrix metalloproteases (MMPs) and their tissue inhibitors into platelets [127]. Nevertheless, the sorting mechanisms appear largely unknown [128]. Few studies have expounded whether changes in the megakaryocyte environment would alter the types and amounts of RNA sorting to platelets [129].

EVs also have the ability to transmit information to platelets horizontally [99]. EVs are membrane-separated subcellular particles containing a variety of biologically active molecules. They are the main messengers of local and systemic intercellular biological information exchange [130], and contain nearly all types of non-protein-coding RNAs (ncRNAs), which can be transferred horizontally between cells regulating gene expression and the malignant phenotype in recipient cells, [131]. The results of deep RNA sequencing showed that the proportion of snRNAs was 25%, accounting for the majority of all short ncRNAs in cells, among which 11% in microvesicles (MVs), and 20% in exosomes [132]. While another study confirmed that the expression level of snRNA RNU6-1 was significantly increased in serum EVs of neuroblastoma patients [133]. Our previous research also reported that TEP U1, U2 and U5 levels were closely correlated between platelet and paired exosomes, indicating that snRNAs might be released from tumors to educate platelets through EVs [134].

### Alterations of snRNAs as cancer biomarker

As shown in Table 4, alterations of snRNAs have been reported in multiple tumors. It was reported in the 1102-case research that three differential snRNAs including RNU1-106 P, RNU6-850 P, and RNU6-529 P were found in pan-adenocarcinomas of the esophagus, stomach, colon, and rectum digestive tract, with potential as the biomarkers for diagnosis and progression monitoring for cancer [135]. Moreover, U2 is one of the most highly-expressed in blood and widely-studied snRNAs as a potential tumor marker [136]. Fragments derived from U2 snRNA (RNU2-1f) were differentially expressed in a variety of tumors, with the upregulation not only in serum [137–141] but also in cerebrospinal fluid [142], serving as the potential diagnostic biomarker. It also acts as the prognostic factor. Its relatively high expression of serum RNU2-1f was closely related to shorter median survival in lung cancer patients [137] and a high risk of recurrence and poor prognosis in ovarian cancer [139] (Table 4).

**Table 4:** Alterations of snRNAs in patients with cancer

Materials	SnRNAs	Aberrant expression	Tumor types	References
tissue	RNU4-1, U3	up-regulated	colorectal carcinoma	[186]
	RNU1-106 P, RNU6-850 P	up-regulated	esophageal adenocarcinoma, stomach adenocarcinoma, colon adenocarcinoma, rectal adenocarcinoma	[135]
	RNU6-529 P	down-regulated		
	RNU6-101 P	up-regulated	esophageal adenocarcinoma	
	RNVU1-4	down-regulated	stomach adenocarcinoma	
serum	RNU2-1f	up-regulated	lung, colorectal, pancreatic, cholangiocarcinoma, ovarian cancers and melanoma	[137, 138, 139, 140, 141]
plasma	U6	up-regulated	breast cancer	[187]
cerebrospinal fluid	RNU2-1f	up-regulated	primary central nervous system lymphoma	[142]
platelet	U1, U2 and U5	down-regulated	lung cancer	[134]

In the previous experiment, we validated whether TEP snRNAs served as the potential biomarkers for lung cancer [134]. TEP U1, U2 and U5 levels were significantly decreased in lung cancer patients, possessing the favorable diagnostic efficiency, especially in early lung cancer. Moreover, their downregulation was correlated with lung cancer progression. It was coincided with previous reports, 99% of differential mRNAs in TEP of untreated lung cancer patients were down-regulated [143]. This might also explain the accumulation of a large number of immature reticulated platelets in the blood of NSCLC patients and the down-regulation of splicing function blocked the maturation of reticulated platelets [95].

## Conclusion and perspective

The bidirectional tumor-platelet interactions are reciprocal and complicated, during which the platelets are educated by tumor and derived bio-substance, and empowered with the potential to identify surrogate biomarker signatures to detect cancer. Multiple studies have shown that platelet-based biomarkers (e.g., count, volume, RNA profile and protein profile) are incorporated into liquid biopsy platforms [144]. As the liquid biopsy tool, platelets are easily isolated and counted and are the second most abundant cell in circulation, thus making them more attractive for clinical applications [2]. Moreover, platelets occupies the short life span (average of 7 days), and more importantly, splicing activity and rapid protein translation, thereby the contents in TEPs are dynamic and transient in response to external stimuli, providing the opportunity to potentially serve as a promising diagnostic, prognostic, and therapeutic tool that enables high specificity and sensitivity in the search for new ways to fight against malignancies [145].

The unique benefits of TEP for cancer detection are exciting, nevertheless, some limitations should be taken into consideration. It has been reported that the same RNA plays different roles between cells and platelets, indicating different splicing mechanisms in platelets from those in cells [119]. It has been observed that cancer cells disrupt normal alternative splicing events to generate specialized splicing isoforms that affect cell function and control cell proliferation and tumorigenesis [146–148]. Although TEPs as a novel biosource for cancer diagnostics are widely recognized, it is generally uninformed about the mechanisms how conformational and compositional changes within the spliceosome determine splicing outcomes [109], which urgently needs further investigation to enable extended and more optimal diagnostics. Besides, there is still a large gap between biomarker discovery and clinical validation and implementation. The simplified, low-cost and standardized methodologies must be developed. For example, the most commonly used method of platelet isolation is low-speed centrifugation, but the protocols quite differ from different researches and laboratories [149]. Therefore, consensus on methods for TEP research of normalization, sample collection, and processing is essential and imperative. Another critical point for the TEP clinical implementation would be to perform clinical utility studies. A dedicated, well-powered, blinded, and population-targeted prospective clinical trial based on TEP platform should be further pursued as other types of liquid biopsies to ensure the clinical value of platelet-related biomarkers including RNA splicing signatures [150]. Collectively, we believe that TEP RNA repertoire and RNA processing machineries including snRNAs will be widely used in cancer diagnosis, treatment and prognosis monitoring in future, bringing great progress to the cancer diagnostics and treatment and warrant further research.

## Data Availability

The data supporting the conclusions of this article will be made available by the authors, without undue reservation.
